# Chylothorax, in the Spotlight of Lymphangioleiomyomatosis

**DOI:** 10.1016/j.opresp.2022.100194

**Published:** 2022-08-01

**Authors:** Pablo Gámez-Baeza, Ana Belén Gámiz-Molina, María López-López, Emilia Navascués-Martínez

**Affiliations:** Servicio de Neumología, Hospital Universitario Clínico San Cecilio, Granada, Spain

Dear Editor,

Lymphangioleiomyomatosis (LAM) is a rare systemic disease which affects approximately 1/400,000 women in childbearing age. It has two forms of presentation: sporadic (S-LAM) or as part of a genetic disease, tuberous sclerosis (TSC-LAM).^1^

It presents non-specific symptoms, such as dyspnea or cough, however, it highlights a greater predisposition to associated complications such as recurrent pneumothorax, chylothorax or association with angiomyolipomas or lymphangioleiomyomas at the abdominal level.^2^

Histopathologically, it is characterized by the proliferation of abnormal smooth muscle cells that accumulate in the peribronchial, perivascular and perilymphatic regions of the lung, causing cystic destruction of the lung parenchyma, whose associated molecular and cellular processes include the inactivation of tuberous sclerosis complex genes, TSC1 and TSC2, and activation of the mammalian target of rapamycin (mTOR) pathway. Although inhibition of the mTOR pathway with drugs such as sirolimus and everolimus has shown benefits in these patients, with stabilization of lung function and improvement in quality of life, discontinuation of treatment results in recurrence of disease progression.^3^

We present the case of a 43-year-old non-smoking woman, with no relevant medical history, who was treated in our emergency department due to progressively increasing dyspnea in the last year, more accentuated in the last month, accompanied by occasional cough without expectoration and atypical chest pain. On examination, she presented good general condition, hemodynamically stable, and basal SaO_2_ of 95%, with bilateral hypophonesis standing out on respiratory auscultation. In the laboratory tests requested, no noteworthy alterations were observed, except for severe hypoxemia with hypocapnia (basal ABG: pH 7.43; pCO_2_ 31 mmHg, pO_2_ 65 mmHg, HCO_3_ 22 mmoL/L, lactic acid 0.7 mmol/L). The imaging tests that were performed are attached below (Fig. 1). The chest X-ray revealed a mild left pleural effusion and the chest AngioCT showed countless pulmonary cysts distributed throughout the lung parenchyma with associated left pleural effusion.

**Fig. 1:**
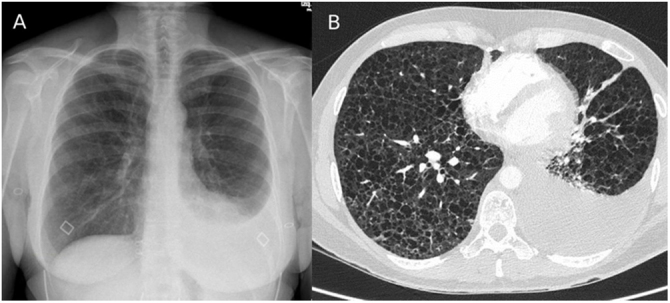
(A) Posteroanterior chest X-ray: Mild left pleural effusion. (B) AngioCT Chest: Countless pulmonary cysts distributed throughout the lung parenchyma with associated left pleural effusion.

In the differential diagnosis, the possibility of lymphangioleiomyomatosis was considered the most likely option, along with lymphoid interstitial pneumonia and histiocytosis X.

A diagnostic-evacuating thoracocentesis was performed and pleural fluid compatible with chylothorax was obtained (milky appearance, triglycerides at 2929 mg/dL and cholesterol less than 20 mg/dL). The patient was admitted to our Pneumology department and a diet with medium-chain triglycerides was started. The autoimmunity study that was carried out (ANA, ENA, RF, anti-CCP and anti-dsDNA) was negative, with an elevated vascular endothelial growth factor (VEGF) of 284 pg/mL (normal < 128.9 pg/mL) and a negative genetic study (TSC1 and TSC2).

During admission, an abdominal MRI was requested, which revealed multiple retroperitoneal lymphangioleiomyomas. In the respiratory function tests, a severe generalized obstruction was detected that caused air trapping and hyperinflation, associating a severely decreased diffusion of CO (DLCOc). FEV1: 46% (1320 mL), FVC: 88% (2940 mL), FEV1/FVC: 45%, MMEF 25–75: 15% (540 mL/s), TLC: 120% (6210 mL), ITGV: 141% (3900 mL), RV: 192% (3240 mL), DLCOc 25%, VA 88% (4430 mL).

With the results obtained, the clinical diagnosis of lymphangioleiomyomatosis was reached, due to the finding of pulmonary cysts, lymphangioleiomyomas and chylothorax in the tests carried out, presenting a compatible clinical context. Treatment with sirolimus was started, maintaining the dose at 1 mg because of the appearance of hypertransaminasemia with a dose of 2 mg, and the patient strictly adhered to the recommended diet. However, the patient was transferred to the thoracic surgery service after clinical worsening and progression of the left chylothorax, requiring the insertion of a pleural drain and initiation of parenteral nutrition. Finally, due to poor control with persistent discharge through the drainage despite thoracic duct embolization, video-assisted thoracoscopy and left pleurodesis were performed, with subsequent hospital discharge after improvement.

When the patient was checked again, liver enzymes had normalized, so the dose of sirolimus was progressively increased until it was maintained at 2 mg. After six months of treatment, a slight improvement in lung function was observed (FEV1 1720 mL – 60% and DLCOc 32%), without recurrence of pleural effusion in the control chest X-ray and with significant clinical improvement. However, in the 6-minute walk test, a total distance of 505 meters was observed, with SaO_2_ < 90% from the first minute, SaO_2_ < 80% from minute 4 and a minimum SaO_2_ of 74%, so the lung transplant unit was contacted for their assessment.

Chylothorax is an exudate-type pleural effusion rich in triglycerides and chylomicrons (which gives it a milky appearance), and it occurs in LAM with an incidence between 7 and 31%. It can be produced by several mechanisms, mainly due to obstruction or rupture of the thoracic duct and it usually recurs if the treatment consists only of chest drainage,^4^ as was the case with our patient. It must be taken into account that it produces a nutritional deficit and the measures to be taken to reduce chyle production consist of taking light chain fatty acids, treatment with octreotide, and in cases of recurrence (what is usual), pleurodesis, pleurectomy, or thoracic duct ligation.^5^ In this clinical case we therefore show the importance of a good control and treatment of the chylothorax, with the aim of avoiding a LAM complication that is not very common.

## Authors’ contributions

All authors have made substantial contributions in each of the following aspects: (1) conception, clinical case design and data acquisition, (2) drafting of the article and critical revision of the intellectual content, (3) final approval of the presented version.

## Informed consent

Informed consent was obtained from the patient's relative for publication of the clinical data and images present in this manuscript.

## Funding

The authors declare that they have not received any fees or funding for the development of the clinical case presented.

## Conflicts of interest

The authors declare that they have no known competing financial interests or personal relationships that could have appeared to influence the work reported in this paper.
